# Enhanced high-energy proton radiation hardness of ZnO thin-film transistors with a passivation layer

**DOI:** 10.1186/s40580-025-00474-5

**Published:** 2025-01-30

**Authors:** Yongsu Lee, Hae-Won Lee, Su Jin Kim, Jeong Min Park, Byoung Hun Lee, Chang Goo Kang

**Affiliations:** 1 Advanced Radiation Technology Institute, Korea Atomic Energy Research Institute, 29 Geumgu-gil, Jeongeup-si, Jeolabuk-do 56212 Republic of Korea; 2 Center for Semiconductor Technology Convergence, Department of Electrical Engineering, Pohang University of Science and Technology, Cheongam-ro 77, Nam-gu, Pohang, Gyeongbuk 37673 Republic of Korea

**Keywords:** Radiation hardening, Proton irradiation, Thin-film semiconductor, ZnO, Passivation

## Abstract

Metal-oxide thin-film semiconductors have been highlighted as next-generation space semiconductors owing to their excellent radiation hardness based on their dimensional advantages of very low thickness and insensitivity to crystal structure. However, thin-film transistors (TFTs) do not exhibit intrinsic radiation hardness owing to the chemical reactions at the interface exposed to ambient air. In this study, significantly enhanced radiation hardness of Al_2_O_3_-passivated ZnO TFTs against high-energy protons with energies of up to 100 MeV is obtained owing to the passivation layer blocking interactions with external reactants, thereby maintaining the chemical stability of the thin-film semiconductor. These results highlight the potential of passivated metal-oxide thin films for developing reliable radiation-hardened semiconductor devices that can be used in harsh space environments. In addition, the relationship between low-frequency noise and defects due to oxygen vacancies was revealed, which can be utilized to improve device reliability.

## Introduction

The demand for electrical systems operating in radiation environments has rapidly increased with the expansion of human space technologies, such as satellites and lunar exploration [1–7]. In radiation environments, the semiconductor circuits in electrical products are susceptible to degradation, malfunction, and permanent failure owing to ionization and direct damage to the crystalline structure by radiation [8–10]. As such, integrated circuits are shielded against external radiation, such as gamma rays and low-energy proton, using thick metal packaging modules based on Al, W, and Ta [11–13]. However, effective shielding against high-energy particle radiation of over 30 MeV is difficult to achieve, prompting additional side effects because of the secondary particles generated as radiation particles collide with the metal shielding layers [14–16]. Furthermore, such metal shielding systems should be minimized to reduce the weight of electrical systems, which is an important factor for space launch vehicles [17]. Therefore, semiconductor products should exhibit radiation hardness to maintain their performance and reliability in high-energy radiation environments.

Conventional Si-based metal–oxide–semiconductor field-effect transistor devices are used in integrated circuits for radiation environments; however, they are prone to degradation and malfunction under high-energy radiation owing to ionization effects that cause severe damage, which can result in permanent failure [18–20]. In addition, damage to bulk semiconductors composed of fine epitaxial structures is a major mechanism for radiation damage and is accelerated with transistor scaling [21]. As such, a fundamental solution is required for the stable operation of semiconductors in harsh radiation environments.

Among next-generation semiconductors, oxide thin-film materials, which exhibit excellent electrical properties, large-area feasibility, mechanical flexibility, and expandability for three-dimensional integration applications, have emerged as promising radiation-hardness semiconductors [22–29]. Unlike epitaxial silicon, the tolerance of the atomic structure of oxide thin-film semiconductors composed of amorphous or polycrystalline materials decreases their susceptibility to crystal changes caused by external radiation. In addition, the nanoscale thickness has the dimensional advantage of minimizing the irradiated cross-sectional area.

The radiation hardness of metal-oxide thin-film transistors (TFTs) has been investigated. Among different radiation types, protons have been mainly used because charged-particle-type radiation is used to comprehensively analyze the effects of radiation in the space environment. Several researchers have focused on improving radiation hardness by adding Sn and Hf, which are metal elements with high O binding energies, to increase the bulk bond strength of metal-oxide semiconductors [22–24, 28–33]. However, the thickness of the channels used was several tens of nanometers, diluting their intrinsic radiation hardness. For thin-film semiconductors with a thickness of several nanometers, the proportion of surface states greatly increases. Therefore, an in-depth analysis of the effect of a passivation layer that can control the surface states exposed to ambient air is required. Further, the radiation hardness against high-energy proton irradiation should be further explored to consider geostationary and deep-space situations.

In this study, the radiation hardness of high-energy protons in ZnO TFTs was investigated. An Al_2_O_3_ passivation layer was introduced to significantly improve the radiation hardness of ZnO TFTs for high-energy protons with energies of up to 100 MeV. In addition, a mechanism for improving the radiation hardness was established based on the invariance of the chemical states of the thin-film semiconductor. Finally, a direction for the development of highly reliable thin-film semiconductors is suggested by revealing the close correlation between the oxygen vacancy state and low-frequency noise.

## Results and discussion

### Fabrication and electrical characteristics of ZnO TFTs

**Fig. 1:**
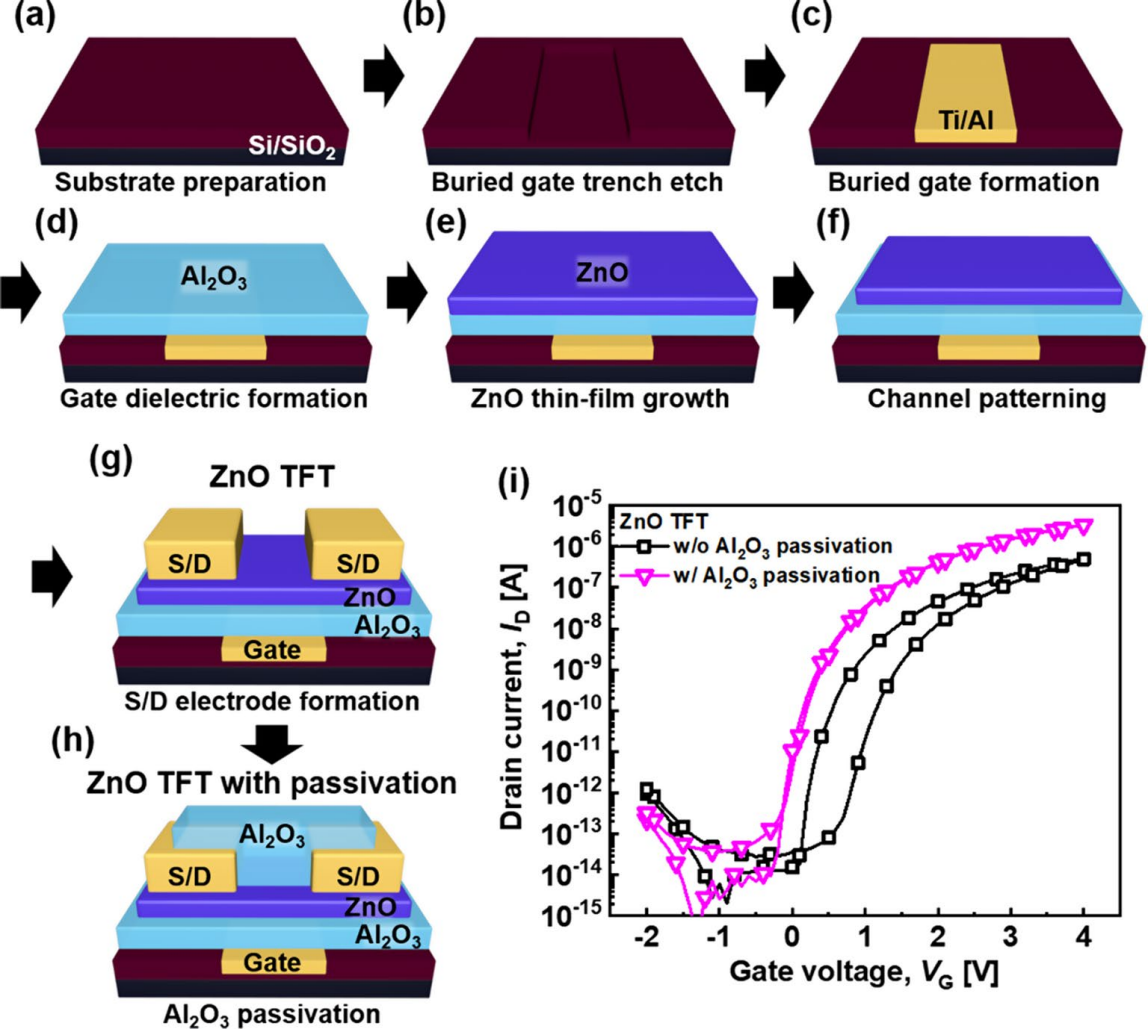
(**a**–**h**) Schematic of the fabrication process of the ZnO TFTs with and without a passivation layer. (**i**) Transfer curves of ZnO TFTs with and without an Al_2_O_3_ passivation layer at V_D_ = 2 V

Figure 1(a–h) show the fabrication process of the ZnO TFT. A Si/SiO_2_ wafer was used as the substrate (Fig. 1a). For the buried gate electrode, SiO_2_ in the gate region was etched by reactive-ion etching to form an oxide trench (Fig. 1b). The buried gate has the advantage of a more uniform electric field distribution than the general bottom gate [34]. The filling metal on the oxide trench is Ti/Al, where Ti and Al were used as the adhesion layer and low-work-function gate metal for n-type thin-film semiconductors, respectively (Fig. 1c). As the gate dielectric, 10 nm Al_2_O_3_ was deposited on the Al gate electrode (Fig. 1d). For the n-type thin-film semiconductor, 3.5 nm ZnO was deposited on the gate dielectric (Fig. 1e). Both films were deposited by atomic layer deposition (ALD), which is the most suitable process for fabricating high-quality large-area integrated circuits at low temperatures with good film quality [35]. The ZnO channel was patterned by photolithography and wet etching (Fig. 1f), and Al metal contact electrodes were formed on the ZnO channel (Fig. 1g). The fabricated ZnO TFT exhibits a staggered structure consisting of the buried bottom-gate and top-contact electrodes with channel width (W) and length (L) of 16 and 12 μm, respectively. The passivation effect was investigated by passivating 10 nm Al_2_O_3_ on the ZnO TFT (Fig. 1h).

Figure 1i shows the electrical characteristics of the ZnO TFTs. The ZnO TFT without an Al_2_O_3_ passivation layer exhibits a typical enhancement-mode n-type TFT curve with a high current-switching property and slight hysteresis. This hysteresis is caused by the additional electron carriers generated by the absorption of O_2_ and desorption of H_2_O molecules at the exposed channel surfaces induced by the electric field of the gate voltage [36]. After Al_2_O_3_ passivation, the hysteresis is dramatically reduced because the passivation layer limits the access of the molecules to the thin-film channel. Furthermore, the threshold voltage (V_th_) shifts to a negative gate voltage (V_G_), and the on-current (I_on_) increases by approximately one order of magnitude. Such behavior is attributed to the reduced defect states on the upper surface of the ZnO channel exposed to ambient air by the passivation layer. Thus, Al_2_O_3_ passivation improved the electrical performance of metal-oxide-semiconductor-based TFTs.

### Effect of high-energy proton irradiation on ZnO TFTs

**Fig. 2:**
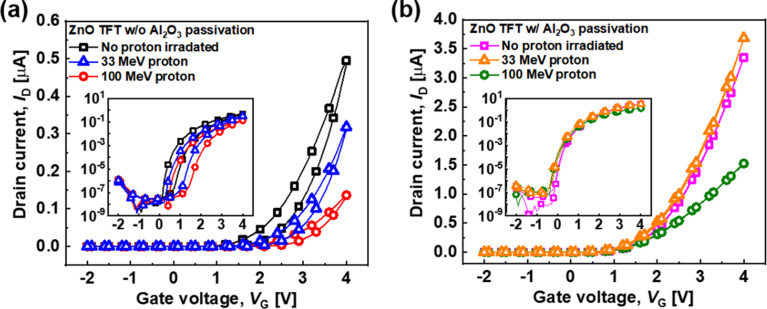
Linear transfer curves of the ZnO TFTs (**a**) without and (**b**) with a passivation layer before and after proton irradiation at V_D_ = 2 V. (inset: log-scale transfer curves)

The ZnO TFTs were irradiated with proton beams with the energy of 33 and 100 MeV, and the devices without and with a passivation layer were electrically characterized; the results are shown in Fig. 2a and b, respectively. I_on_ is defined as the maximum current of each transfer curve, and V_th_ is extracted using the constant-current method at 10^− 10^ A. The hysteresis is defined as the difference of V_th_ between the forward and reverse currents from each transfer curve. The subthreshold swing (SS) is defined as the required V_G_ change to increase the drain current (I_D_) by one order of magnitude in the subthreshold region of each transfer curve, as shown in Eq. (1):

1 $$\:\text{S}\text{S}=\:\frac{\partial\:{V}_{G}}{\partial\:{\text{log}}_{10}{I}_{D}}$$

Field-effect mobility (µ_FE_) is calculated by the following equation:

2 $$\:{{\mu\:}}_{FE}=\:{\left.\frac{L}{W}\frac{{g}_{m}}{{C}_{ox}{V}_{D}}\right|}_{max}$$

where g_m_ is the transconductance, C_ox_ is the gate dielectric capacitance per unit area, and V_D_ is the drain voltage. The values of these parameters before and after proton irradiation are summarized in Table 1.

**Table 1:** Comparison of the electrical characteristics of the ZnO TFTs

Device type	Proton energy [MeV]	On current [µA]	Subthreshold swing [mV/dec]	Field-effect mobility [cm^2^/V·s]	V_th_ [V]	Hysteresis [V]
ZnO	No proton	0.44 ± 0.05	146.7 ± 3.4	0.42 ± 0.04	0.70 ± 0.17	0.63 ± 0.10
	33	0.34 ± 0.07	150.5 ± 2.1	0.37 ± 0.07	0.83 ± 0.05	0.70 ± 0.08
	100	0.23 ± 0.10	162.6 ± 12.0	0.26 ± 0.11	0.92 ± 0.10	0.73 ± 0.08
ZnO/Al_2_O_3_	No proton	3.09 ± 0.79	122.3 ± 5.7	1.52 ± 0.49	0.02 ± 0.10	0.07 ± 0.05
	33	3.26 ± 0.78	136.7 ± 5.5	1.56 ± 0.40	−0.06 ± 0.07	0.09 ± 0.04
	100	1.86 ± 0.46	148.4 ± 7.7	0.96 ± 0.23	−0.07 ± 0.04	0.08 ± 0.04

For the ZnO TFTs without passivation, the electrical performance of the devices was degraded by high-energy proton irradiation. Specifically, after proton irradiation, V_th_ positively shifted, and I_on_, SS, µ_FE_, and hysteresis values decreased. These tendencies became more prominent as the irradiated proton energy increased. In contrast, different proton irradiation effects were noted for the ZnO TFT with a passivation layer. First, V_th_ slightly shifted toward a negative V_G_, which is the opposite of that of the ZnO TFT without passivation. Such behavior is explained by the total ionizing dose (TID) effect, which indicates the accumulation of trapped hole carriers on an insulator due to ionization by irradiation. This effect becomes dominant in ZnO TFTs with passivation owing to the additional Al_2_O_3_ insulator layer on the channel [37]. In addition, the electrical performances of the passivated devices were less degraded after proton irradiation than those of the unpassivated devices. Therefore, passivated ZnO TFTs exhibited radiation hardness in terms of the electrical performance compared to unpassivated TFTs. Some of the electrical performances, such as I_on_ and µ_FE_, were slightly degraded with proton irradiation at 100 MeV, suggesting the damage induced by the higher energy proton irradiation even with the passivation layer.

### Electrical stress test before and after proton irradiation

**Fig. 3:**
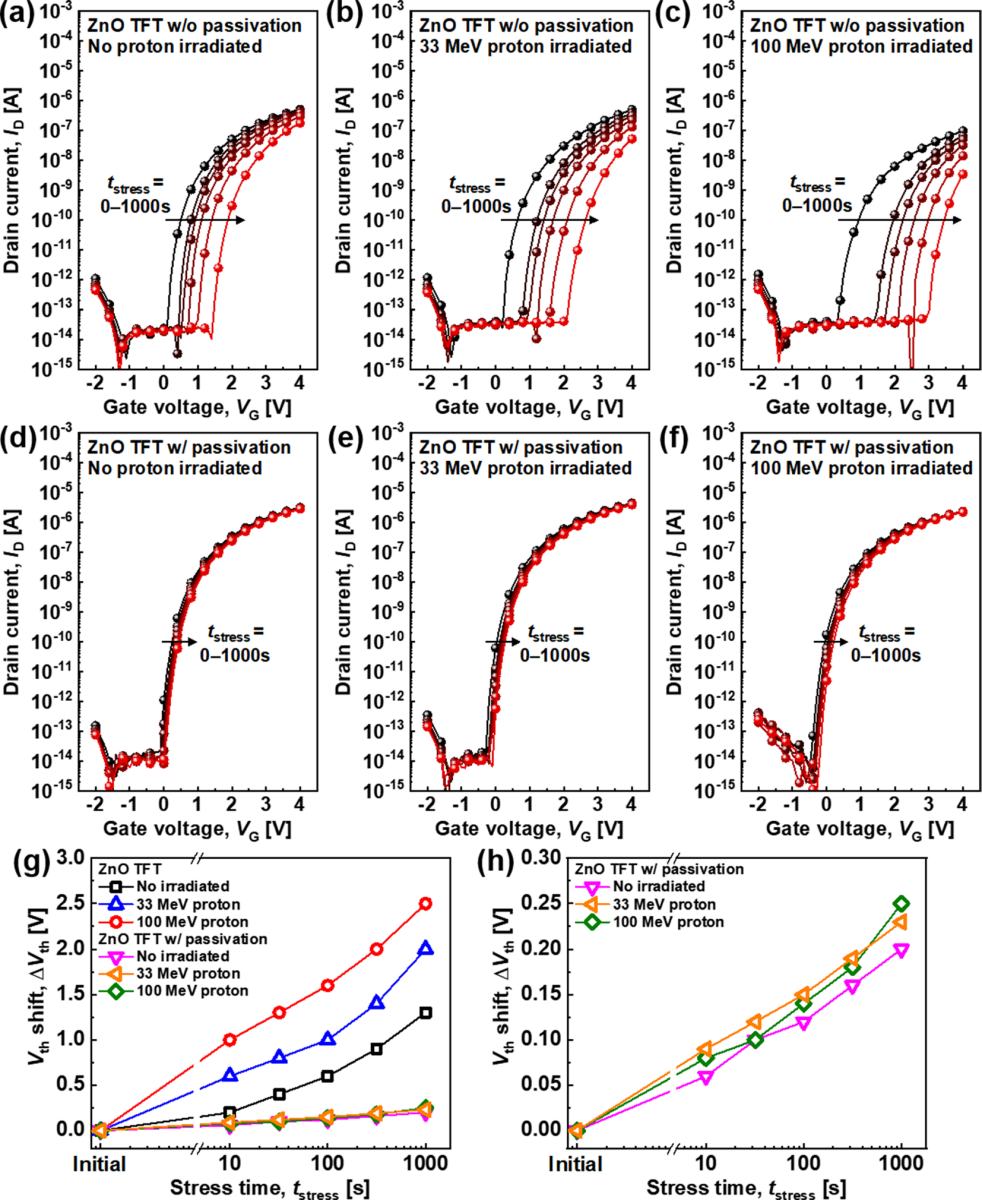
Stress measurement of the ZnO TFTs without passivation (**a**) before and after proton irradiation at (**b**) 33 MeV and (**c**) 100 MeV. Stress measurement of the ZnO TFTs with passivation (**d**) before and after proton irradiation at (**e**) 33 MeV and (**f**) 100 MeV. The stress condition is V_D_ = V_G_ = 4 V for 0 (initial), 10, 32, 100, 316, and 1000 s. Extracted ΔV_th_ from the stress measurements of (**g**) all devices and (h) ZnO TFTs with passivation only

For reliability analysis, a voltage-bias stress test was conducted, whereby the positive V_G_ and V_D_ stresses were biased, and the devices were electrically characterized. The results are shown in Fig. 3. The V_th_ of ZnO TFTs without passivation shifted toward positive V_G_ values with increasing stress time (t_stress_) owing to electron carrier trapping on the defect sites in the ZnO bulk and ZnO/gate dielectric interface and ZnO channel surfaces (Fig. 3a). After proton irradiation, V_th_ further shifted towards positive values, denoting further degradation of the reliability of the ZnO TFT without passivation as the proton energy increased (Fig. 3b–c). Similarly, V_th_ shifted toward a positive V_G_ for the passivated ZnO TFT; however, the change is smaller than that of the unpassivated devices. This is consistent with the results shown in Fig. 1i, where the electron carrier trapping on the ZnO channel surfaces was dramatically reduced by Al_2_O_3_ passivation (Fig. 3d). After proton irradiation, the V_th_ of the passivated ZnO TFTs still shifted slightly, indicating that the passivation layer improved the radiation hardness in terms of the reliability of the metal-oxide TFTs (Fig. 3e–f).

The difference of V_th_ (ΔV_th_) according to the t_stress_ of all stressed ZnO TFTs was extracted and compared, as shown in Fig. 3g. All ΔV_th_ of ZnO TFTs increases as t_stress_ increases. ΔV_th_ of the ZnO TFT without passivation increased as the proton energy increased, indicating poor radiation hardness to high-energy proton beams, whereas ΔV_th_ of the passivated ZnO TFT is reduced to 1/10 of that of the unpassivated devices, indicating improved reliability and radiation hardness. Further, the passivated ZnO TFTs exhibited a slight increase in ΔV_th_ after proton irradiation, denoting that the proton irradiation accelerated the generation of defects at the bulk and interface between the ZnO channel and Al_2_O_3_ dielectric (Fig. 3h). Nonetheless, minimal damage is noted because of the nanoscale material properties of the thin-film semiconductors.

### Chemical analysis of ZnO TFT under proton irradiation

**Fig. 4:**
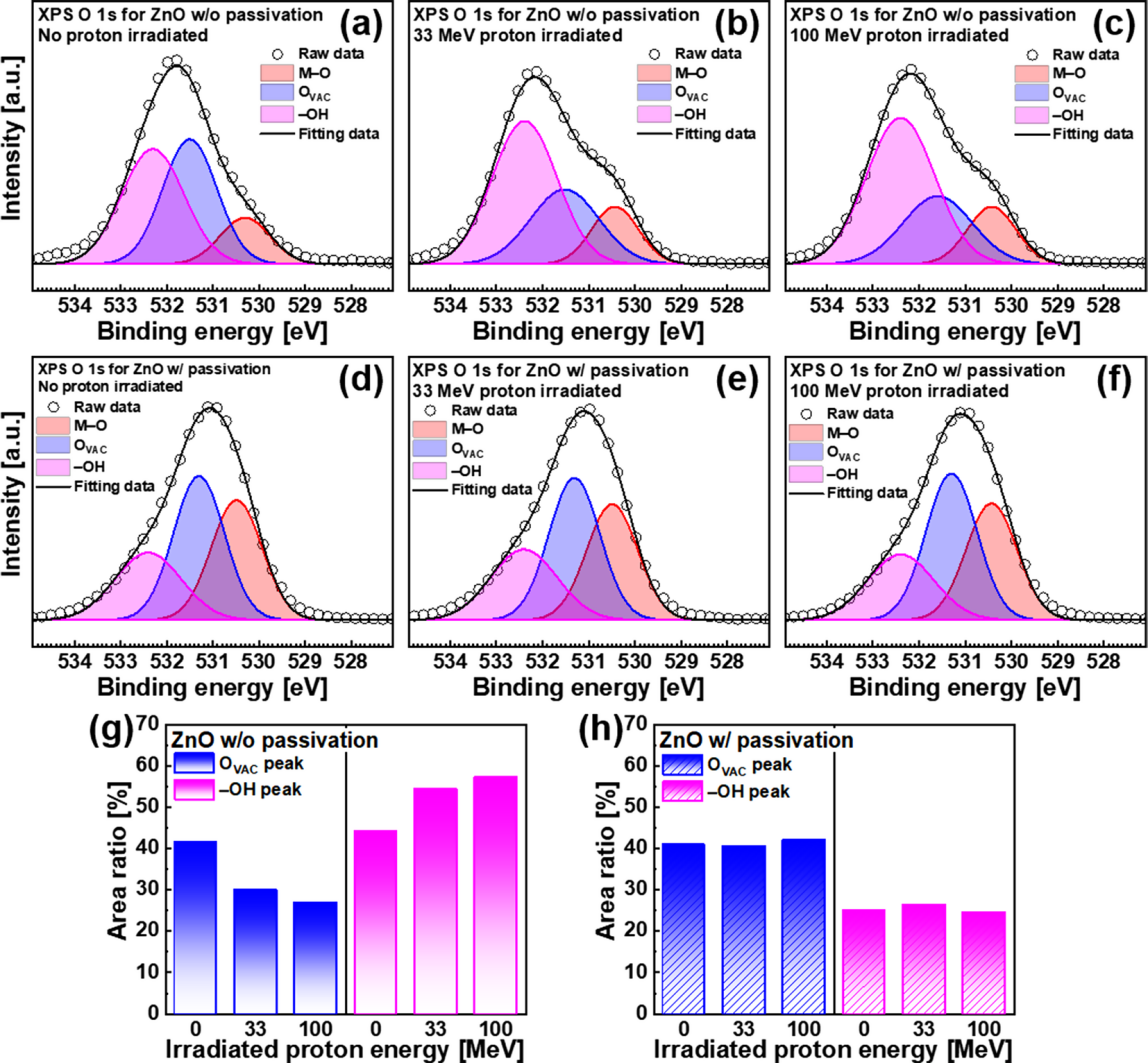
XPS O 1s spectra of the ZnO without passivation (**a**) before and after proton irradiation at (**b**) 33 MeV and (**c**) 100 MeV and with passivation (**d**) before and after proton irradiation at (**e**) 33 MeV and (**f**) 100 MeV. Extracted O_vac_ and –OH peak ratio from the XPS O 1s spectra of the ZnO (**g**) without and (**h**) with passivation

The principle for the enhanced radiation hardening of the ZnO TFT after Al_2_O_3_ passivation was verified by the X-ray photoelectron spectroscopy (XPS). Figure 4a–f show the O 1s XPS spectrum of the ZnO channel, which is a composite of three Gaussian function components representing different chemical states. The low binding energy at 530.5 ± 0.1 eV is attributed to the O^2−^ ions surrounded with Zn^2+^ ions that are completely bonded to adjacent O^2−^ ions (M–O peak). The middle binding energy at 531.4 ± 0.1 eV is ascribed to the O^2−^ ions near the Zn^2+^ ions with oxygen vacancies (O_vac_ peak). The high binding energy at 532.4 ± 0.1 eV is related to the hydroxyl group species formed by the adsorption of H_2_O or O_2_ on the surface of the ZnO layer (–OH peak) [38].

For the unpassivated ZnO TFT, a small M–O peak is observed owing to the amorphous structure of the ZnO layer deposited at a low temperature (~ 100 °C) and small thickness (~ 3.5 nm) (Fig. 4a). When ZnO was exposed to ambient air, several –OH groups were generated on its surface. After proton irradiation, the intensity of the –OH peak increases and that of the O_vac_ peak decreases, prompting the substitution of O_vac_ by the –OH groups owing to reactions with external H_2_O mediated by proton energy (Fig. 4b and c). For the ZnO TFT with an Al_2_O_3_ passivation layer, a large M–O peak and small –OH peak are observed, which are considered to be the result of the replacement of –OH groups with the M–O bond during the ALD process for the Al_2_O_3_ passivation layer (Fig. 4d). The low number of –OH bonds is one of the main reasons for the improved electrical performance of the ZnO TFT after passivation. The XPS results of the ZnO TFT with passivation were difficult to observe after proton irradiation (Fig. 4e–f).

For detailed analysis, the area ratios of each component were compared. Figure 4g shows the area ratio of O_vac_ and –OH peaks for ZnO without passivation before and after proton irradiation. The O_vac_ peak of ZnO without passivation decreases rapidly as the proton energy increases, which is consistent with the V_th_ shift of the ZnO TFTs without passivation (Fig. 2a) because ionized oxygen vacancy states are considered n-type shallow dopants in metal-oxide thin-film semiconductors [39]. In addition, the intensity of the –OH peak increases rapidly as the proton energy increases, indicating the absorption of more H_2_O molecules on the ZnO surface, which causes the larger hysteresis and ΔV_th_ shown in Figs. 2a and 3a–c. The area ratio of the O_vac_ and –OH peaks of the passivated ZnO TFT before and after proton irradiation is shown in Fig. 4h, in which almost no changes are noted. This is consistent with the excellent radiation hardness properties of the ZnO TFTs with passivation, with maintained electrical performance before and after proton irradiation, as shown in Fig. 2b. Despite the invariability of the chemical states, some differences in the electrical characteristics of passivated ZnO TFTs after proton irradiation require further explanation. This could be interpreted as other physical phenomena that are not detected by chemical analysis, such as V_th_ shifts by the TID effect on Al_2_O_3_ dielectrics and electrical degradation by the displacement damage (DD) effects in the ZnO channel and/or Al_2_O_3_ dielectrics owing to the higher proton irradiation energy, which results in cumulative nonionizing damage to the semiconductor lattice owing to the impact of irradiation [8, 9]. Nevertheless, it is emphasized again that these phenomena are minimized due to the low-dimensional material properties of the metal-oxide thin-film semiconductors.

### Low-frequency noise characteristics of ZnO TFTs

**Fig. 5:**
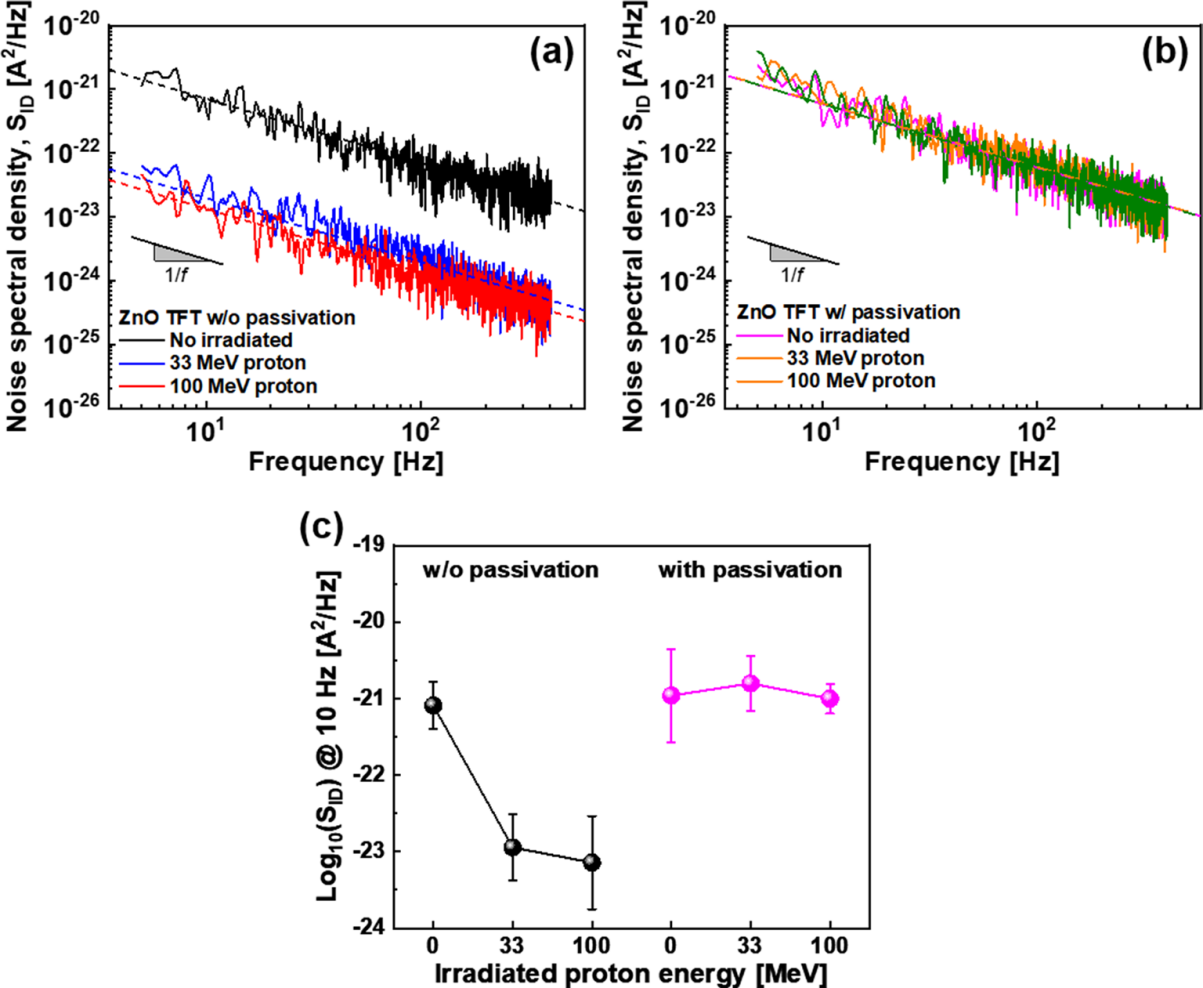
Low-frequency noise spectral densities of the ZnO TFTs (**a**) without and (**b**) with passivation before and after proton irradiation. (**c**) Extracted noise spectral densities at a frequency of 10 Hz

The low-frequency noise of the ZnO TFTs before and after proton irradiation is elucidated. In electrical devices, low-frequency noise is a reliability indicator because high noise disrupts the consistency of the electrical characteristics of the devices and causes signal errors, resulting in distorted information transmission [40]. Flicker noise, also called 1/f noise, which is the most common noise in electrical devices, is inversely proportional to the frequency and is caused by fluctuations in the carrier mobility or number of carriers owing to the interactions with defects [41]. Figure 5a and b show the low-frequency noise spectral densities (S_ID_) before and after proton irradiation for the ZnO TFTs with and without passivation, respectively. A low-frequency noise with a slope of − 1, denoting flicker noise, is observed. For the unpassivated ZnO TFTs, the degradation of noise characteristics is expected with an increase in defects after proton irradiation; however, interestingly, the flicker noise is significantly reduced after proton irradiation. This is inferred to be due to chemical changes caused by proton irradiation. On the other hand, no significant change in the noise characteristics before and after proton irradiation is observed for the passivated ZnO TFTs, confirming the excellent radiation hardness of the ZnO TFTs with passivation.

Figure 5c shows a S_ID_ comparison at 10 Hz, indicating that the noise of the unpassivated ZnO TFTs after proton irradiation is approximately two orders of magnitude lower than that of the other cases. This result is similar to the trends of the O_vac_ peak derived by XPS, as shown in Fig. 4g–h; similar area ratios of O_vac_ peaks are observed for ZnO with and without passivation before proton irradiation. Moreover, minimal changes in the area ratios of O_vac_ peaks are observed among the passivated ZnO layers. At last, the area ratios of O_vac_ peaks decreased rapidly after proton irradiation in the unpassivated ZnO TFTs. These three tendencies are consistent with the noise trend shown in Fig. 5c. Such correlation is interpreted as the high contribution of oxygen vacancies to the low-frequency noise. In fact, oxygen vacancies not only function as shallow donors but also as deep-level defect sites in the metal-oxide semiconductor band structure, so they are expected to cause fluctuations in the number of carriers, thereby increasing the noise in the devices [39]. Note that hydroxyl groups, which are considered defects that cause large hysteresis and bias instability, are expected to increase noise. In fact, hydroxyl groups act as space charges in the ZnO channel, which can cause fluctuations in the mobility of carriers and increase noise. In contrast, they also passivate defects originating from oxygen vacancies, which can contribute to noise reduction [42]. Consequently, the impact of hydroxyl groups is minimal when comparing the hydroxyl group ratios and noise levels of the ZnO TFTs with and without passivation before proton irradiation, and therefore, further improvement in noise characteristics through oxygen vacancy engineering of the metal-oxide thin-film semiconductors is suggested.

## Conclusions

The fabrication of ZnO TFTs with an Al_2_O_3_ passivation layer and increased radiation hardness against high-energy proton irradiation was demonstrated. The electrical performance of metal-oxide TFTs easily degraded owing to interactions with external reactants in ambient air mediated by high-energy protons; however, the introduction of a passivation layer prevented chemical reactions and maintained the chemical stability of the ZnO channel and consequently, achieved the radiation hardness of the TFT device. Although further analysis of the physical degradation factors due to high-energy proton radiation is required, these results highlight the potential for developing stable and radiation-hardened devices for operation in harsh space environments using passivated metal-oxide thin films. Moreover, further optimization of oxygen vacancy engineering can improve the reliability of metal-oxide TFTs, such as low-frequency noise.

## Methods

Figure 1a–h show a schematic of the fabrication process of the ZnO TFTs. A Si/SiO_2_ wafer was used as a substrate, which was cleaned via sonication for 5 min each using acetone, methanol, and distilled water. A buried gate electrode pattern was formed by etching a 70 nm SiO_2_ trench using I-line photolithography and reactive-ion etching using Ar and CF_4_ plasmas. Before removing the photoresist (PR) during photolithography, 10/60 nm Ti/Al metal layers were deposited using an e-beam evaporator in a high-vacuum chamber (~ 10^6^ Torr). After filling the oxide trench with metals, the PR was lifted with redundant metals by sonification in acetone, and the residual metal at the boundary of the buried gate pattern was removed by chemical–mechanical polishing. Subsequently, 10 nm Al_2_O_3_ was deposited as a gate dielectric by ALD at 200 °C. The Al and O precursors were trimethylaluminum and H_2_O were used as the Al and O precursors, respectively. ZnO, an n-type channel material, was deposited by a UV/ozone treatment for 20 min on top of Al_2_O_3_ to form a hydrophilic gate dielectric surface. Subsequently, a 3.5 nm ZnO layer was deposited by ALD at 100 °C with diethylzinc and H_2_O precursors. A ZnO channel pattern was formed using photolithography and wet etching with a diluted HCl solution. For the metal-contact electrode, 70 nm Al metal was deposited, followed by the lift-off process. For the Al_2_O_3_ passivation layer, a 10 nm Al_2_O_3_ layer was deposited by ALD at 150 °C on the ZnO TFT.

The fabricated ZnO TFTs were irradiated using high-energy proton beams at the Korea Multipurpose Accelerator Complex (KOMAC). The irradiated proton beam energies were 33 and 100 MeV, and the total fluence for both energy conditions was ~ 5 × 10^9^ cm^2^.

The fabricated devices were electrically characterized using a semiconductor parameter analyzer (Keithley 4200) at 25 °C in ambient air. For low-frequency noise characterization, a low-noise current preamplifier (Stanford Research Systems, SR 570) and a dynamic signal analyzer (Agilent 35670 A) were used. For statistical analysis, more than 20 devices were electrically characterized. The chemical states of the ZnO layers were characterized by XPS (NEXSA, Thermo Fisher Scientific Inc., USA) with an Al Kα excitation source at the GIST Advanced Institute of Instrumental Analysis (GAIA). All XPS spectra were calibrated to a C 1s electron peak (284.8 eV), and the O 1s electron peaks of ZnO were analyzed. The XPS spectra of the passivated ZnO were measured after etching the Al_2_O_3_ passivation layer with Ar-ion plasma.

## Data Availability

The datasets used and/or analysed during the current study are available from the. corresponding author on reasonable request.
